# Rac inhibition as a novel therapeutic strategy for EGFR/HER2 targeted therapy resistant breast cancer

**DOI:** 10.1186/s12885-021-08366-7

**Published:** 2021-06-01

**Authors:** Luis D. Borrero-García, Maria del Mar Maldonado, Julia Medina-Velázquez, Angel L. Troche-Torres, Luis Velazquez, Nilmary Grafals-Ruiz, Suranganie Dharmawardhane

**Affiliations:** Department of Biochemistry, School of Medicine, University of Puerto Rico Medical Sciences Campus, San Juan, Puerto Rico

**Keywords:** Therapy resistance, Breast cancer, Tyrosine kinase inhibitors (TKIs), Rac inhibitors, EHop-016, MBQ-167

## Abstract

**Background:**

Even though targeted therapies are available for cancers expressing oncogenic epidermal growth receptor (EGFR) and (or) human EGFR2 (HER2), acquired or intrinsic resistance often confounds therapy success. Common mechanisms of therapy resistance involve activating receptor point mutations and (or) upregulation of signaling downstream of EGFR/HER2 to Akt and (or) mitogen activated protein kinase (MAPK) pathways. However, additional pathways of resistance may exist thus, confounding successful therapy.

**Methods:**

To determine novel mechanisms of EGFR/HER2 therapy resistance in breast cancer, gefitinib or lapatinib resistant variants were created from SKBR3 breast cancer cells. Syngenic therapy sensitive and resistant SKBR3 variants were characterized for mechanisms of resistance by mammosphere assays, viability assays, and western blotting for total and phospho proteins.

**Results:**

Gefitinib and lapatinib treatments reduced mammosphere formation in the sensitive cells, but not in the therapy resistant variants, indicating enhanced mesenchymal and cancer stem cell-like characteristics in therapy resistant cells. The therapy resistant variants did not show significant changes in known therapy resistant pathways of AKT and MAPK activities downstream of EGFR/HER2. However, these cells exhibited elevated expression and activation of the small GTPase Rac, which is a pivotal intermediate of GFR signaling in EMT and metastasis. Therefore, the potential of the Rac inhibitors EHop-016 and MBQ-167 to overcome therapy resistance was tested, and found to inhibit viability and induce apoptosis of therapy resistant cells.

**Conclusions:**

Rac inhibition may represent a viable strategy for treatment of EGFR/HER2 targeted therapy resistant breast cancer.

**Supplementary Information:**

The online version contains supplementary material available at 10.1186/s12885-021-08366-7.

## Background

Aggressive breast cancers overexpress Epidermal Growth Factor Receptor (EGFR) family members where ~ 25% of breast cancer patients overexpress human epidermal growth factor receptor 2 (HER2) and ~ 15% overexpress the EGFR1 isoform [1]. EGFR/HER2 overexpression in breast cancer increases breast cancer malignancy by upregulated cancer cell survival, invasion and metastasis, maintenance of stem cell-like tumor cells, and resistance to targeted therapies [2–6]. Therefore, a number of EGFR- and HER2-targeted therapeutics has been developed, and these include small molecules that inhibit the tyrosine kinase domain of the EGFR such as gefitinib (EGFR1) and lapatinib (EGFR1 and HER2) [1, 7, 8]. However, the effectiveness of EGFR tyrosine kinase inhibitors (TKI) s in the clinic has been greatly impaired by the development of de novo or acquired resistance [9–11]. Specifically, trials with gefitinib in breast cancer resulted in poor clinical response indicating that intrinsic resistance to gefitinib, and therefore, to TKIs, is common in breast cancer [12, 13]. Similarly, the initial success of lapatinib, which was developed as an ATP-competitive reversible EGFR/HER2 inhibitor, has also been marred by intrinsic and acquired therapy resistance [14, 15]. Consequently, it is crucial to elucidate the mechanisms of EGFR/HER2 therapy resistance, and to develop targeted strategies to reverse such resistance.

Several mechanisms of acquired resistance to TKIs have been reported, including EGFR gene mutations [16], activation of the phosphoinositide 3-kinase (PI3K)/Akt/mammalian target of rapamycin (mTOR) pathway and the Ras/MAPK pathway [17], as well as epithelial to mesenchymal transition (EMT), where acquisition of cancer stem cell-like phenotypes is associated with resistance to TKIs [10, 18–20]. Metastasis, when the cancer cells undergo EMT and migrate to establish secondary tumors at distant vital sites, remains the major cause of death from breast cancer [5]. Recent studies have shown that therapy resistant breast cancer cells possess more mesenchymal and stem cell-like properties and invade the circulatory system using migratory and invasive properties. Once in the circulatory system, the therapy resistant cells can circulate in the blood or lie dormant in the bone marrow and distant organs, while retaining the capacity for self-renewal [21–23]. Therefore, understanding the mechanisms of resistance leading to the acquisition of EMT and migratory and stem cell-like properties is highly relevant for effective breast cancer cure.

To elucidate novel mechanisms and therapeutic strategies to overcome EGFR/HER2 therapy resistance, we created syngenic SKBR3 human breast cancer cell variants resistant to gefitinib (anti-EGFR) or lapatinib (anti-EGFR/HER2). Therapy resistant variants exhibit a more aggressive mesenchymal phenotype with elevated viability/apoptosis and stem cell like activity, associated with increased expression and activity of the Rho GTPase Rac. Rac is a critical molecular switch activated by EGFR/HER2 signaling to regulate cell proliferation, survival, and migration, and thus EMT and metastasis [24–32]. Consequently, Rac plays a significant role in resistance to EGFR/HER+ breast cancer by acting downstream of EGFR/HER2 therapy resistance mechanisms such as Ras/MAPK and PI3-K/Akt signaling [33–43]. Herein, we demonstrate the potential for Rac inhibitors as targeted therapeutics for EGFR/HER2 therapy resistant breast cancer.

## Methods

### Cell culture

Metastatic human breast cancer cells SKBR3 (American Type Culture Collection) and metastatic cancer cell line MDA-MB-435 (kind gift of Dr. Danny Welch) were maintained in complete culture medium: Dulbecco’s modified Eagle’s medium (Invitrogen) supplemented with 10% fetal bovine serum (Invitrogen) at 37 °C in 5% CO_2_. Gefitinib (Gef.R) and lapatinib resistant (Lap. R) variants were created from these EGFR/HER2 (+) gefitinib and lapatinib sensitive SKBR3 cells by exposing the sensitive cells to a range of concentrations up to 0.5 μM for ~ 6 months. The cells that survived at concentrations > 0.1 μM were selected as resistant variants.

### Cell viability

The CellTiter 96 Non-Radioactive Assay (Promega) was used according to manufacturer’s instructions. Briefly, cells were seeded in a 24 well plate and treated for 48 h with vehicle, gefitinib, lapatinib, trastuzumab, and (or) EHop-016 or MBQ-167 at the indicated concentrations. After incubation, the MTT (3-(4,5-dymethyl thiazol-2-yl)-2,5-diphenyl tetrazolium bromide) reagent was added to the plate (40 μL/well). The plates were incubated for 4 h at 37 °C, followed by the addition of stop solution, and the plates were incubated to facilitate solubilization of formed formazan salts. The absorbance was measured at 570 nm using a microplate reader. Fold resistance for therapy resistant cell lines was quantified, as described in [44], by the ratio of the half maximal inhibitory concentration (IC_50_) of the therapy resistant cell line by the IC_50_ of the therapy sensitive cells.

### Caspase assay

Apoptosis was analyzed by the Caspase-Glo 3/7 activity assay (Promega) as described by the manufacturer. Briefly, cells were seeded in a 96 well plate and treated for 48 h. Luminogenic caspase-3/7 substrate containing a DEVD sequence was added and incubated for 1 h. The luminescence was measured by a plate-reading luminometer.

### Western blotting

Therapy sensitive and resistant variants were lysed and Western blotted using routine procedures. Briefly, equal total protein amounts from cell lysates were run on SDS-PAGE gels and Western blotted using specific antibodies against EGFR, pEGFR, HER2, pHER2, Integrin β3, Nanog, CD133, AKT, pAKT, MAPK, pMAPK and Rac. Anti-β-actin was used for normalization. The integrated density of positive bands of total and phospho EGFR/HER2 were quantified using Image J software, as per routine laboratory protocols [45].

### Mammosphere assay

A mammosphere assay was performed to determine cancer stem cell-like activity, as described in [46]. SKBR3 cells were seeded in ultra-low attachment plates (Corning) at a density of 500 cells/well in serum-free mammary epithelium basal medium (Lonza) supplemented with 1% penicillin/streptomycin (Lonza), B27 supplement minus vitamin A (50X, Gibco), 5 μg/mL insulin (Gibco), 1 μg/mL hydrocortisone (Sigma), 20 ng/mL EGF, and 20 ng/mL fibroblast growth factor (Sigma). Mammospheres were counted using an inverted microscope after 4 days of incubation in 37 °C, 5% CO_2_. Mammosphere forming efficiency (MFE) was calculated as the number of mammospheres divided by the number of cells seeded per well and is expressed as a percentage.

### Rac activation assay

Rac activity was analyzed from SKBR3 sensitive and resistant cell lysates by pull-down assays. The P21-binding domain (PBD) of PAK 1 was used to isolate active GTP-bound Rac, as described previously [47]. Active and total Rac GTPases were separated in a 12% SDS-PAGE gel and identified by Western blotting using Rac specific antibodies (Cell Signaling Technology, Inc).

### Statistical analysis

Statistical comparisons between therapy sensitive and resistant cell lines for SKBR3 cells resistant to gefitinib or lapatinib were conducted by Student’s T test using GraphPad Prism 6. Differentially expressed genes and proteins were selected at > 1.5-fold expression, statistical significance of *p* < 0.05.

## Results

### Development of therapy resistant cell variants

**Fig. 1:**
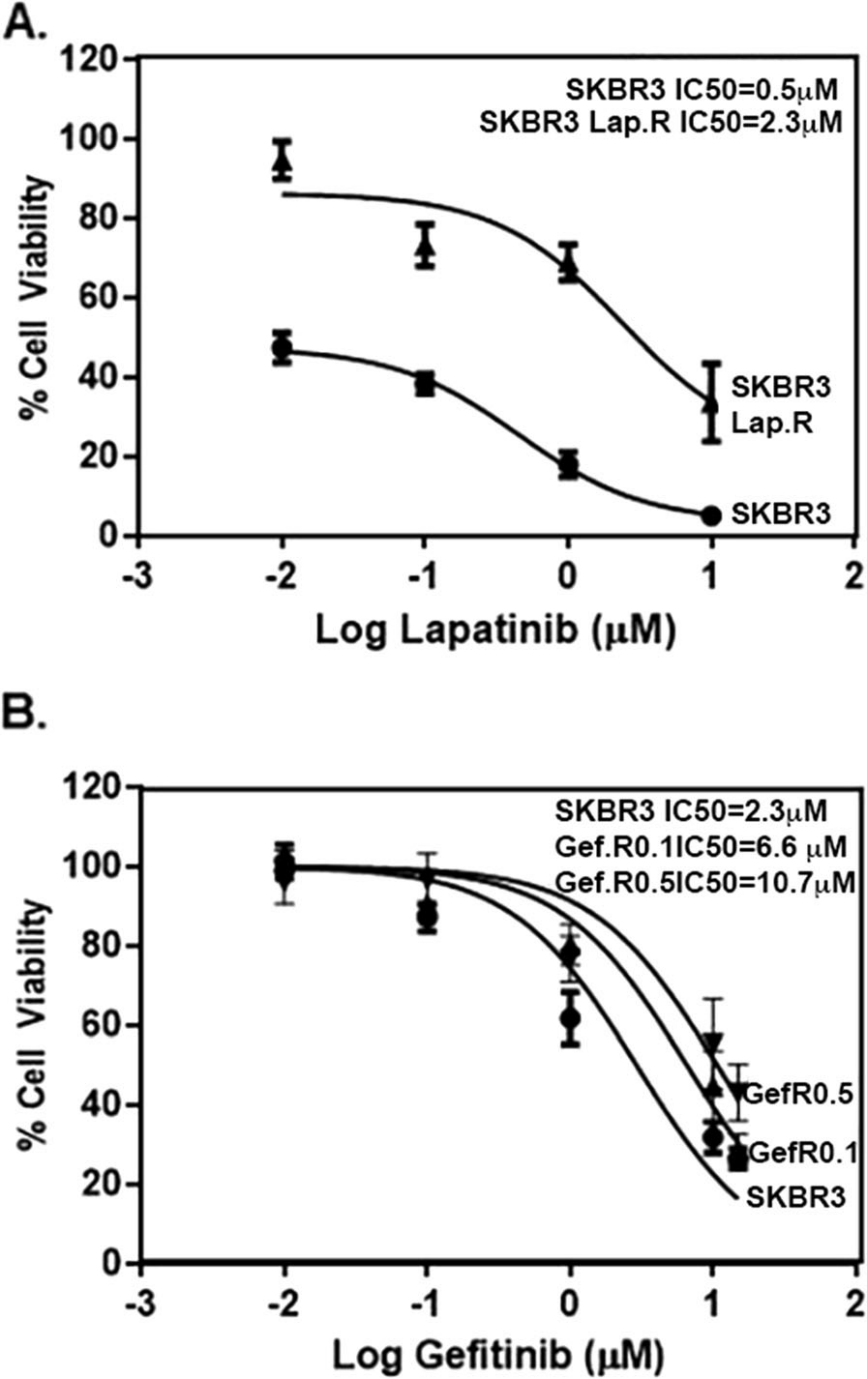
Viability of therapy sensitive and resistant variants in the presence of TKIs. **a** SKBR3 therapy sensitive cells, and variant resistant to 0.1 μM lapatinib, **b** SKBR3 therapy sensitive and variants resistant to 0.1 gefitinib or 0.5 μM gefitinib, were subjected to a MTT cell viability assay to determine the IC_50_ by exposing the cells to different concentrations of TKIs gefitinib and lapatinib. % Cell viability in response to gefitinib or lapatinib is shown for the therapy sensitive and resistant variants. *N* = 4 ± SEM

SKBR3 therapy sensitive EGFR and HER2 positive human breast cancer cells were created following exposure of the cells to gefitinib (0.1 or 0.5 μM) or lapatinib (0.1 μM). After 6 months of selection, the fold resistance was quantified as described in [48], using cell viability as a measure of resistance. Previous studies have established that a range of 2 to 5-fold resistance is required for a therapy resistant cell line to be considered clinically relevant. Cells that reach a fold resistance higher than 5-fold are designated as high laboratory-level resistant, and are useful for studies on mechanisms of resistance. The IC_50_s for viability of the therapy resistant cell lines were divided by the IC_50_ of the therapy sensitive cell line to obtain the fold resistance (Fig. 1). SKBR3 gefitinib resistant (Gef.R) cells at 0.1 μM, and lapatinib resistant (Lap.R) cells at 0.1 μM, demonstrated a fold resistance of 2.3 and 4.6 respectively, whereas Gef. R cells resistant to 0.5 μM gefitinib gave a fold resistance of 3.7. Therefore, the therapy resistant cell lines demonstrated clinically relevant fold resistance and were eligible for further investigation of the mechanisms of resistance.

### EGFR/HER2 activities in therapy resistant breast cancer cells

**Fig. 2:**
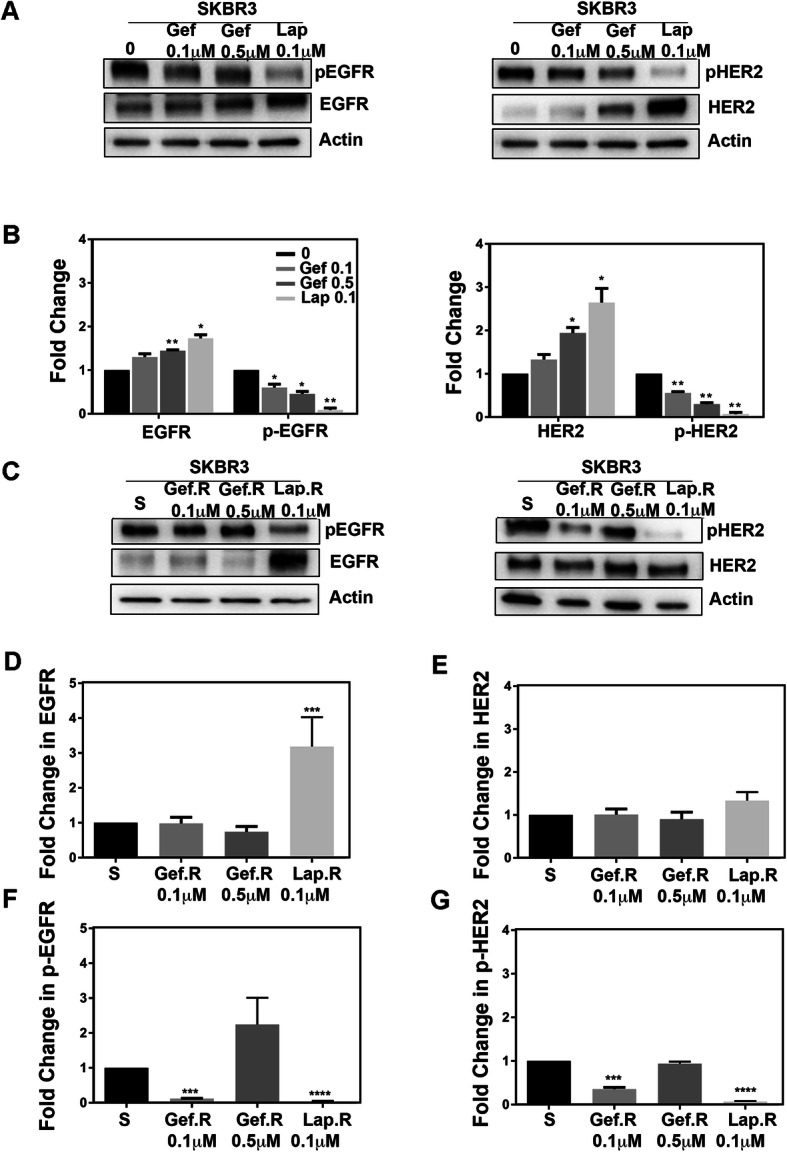
EGFR and HER2 expression and phosphorylation in therapy sensitive and resistant variants. SKBR3 therapy sensitive or resistant (Gef.R 0.1 μM, Gef. R 0.5 μM and Lap. R 0.1 μM) cells treated with gefitinib or lapatinib for 24 h were lysed and western blotted for total and active (phospho) EGFR and HER2. **a** Representative western blots for pEGFR/EGFR (left) and pHER2/HER2 (right), with actin as a loading control, for cells treated with gefitinib or lapatinib for 24 h. **b** Fold change in EGFR and HER2 expression and phosphorylation for the therapy sensitive SKBR3 cells from positive bands quantified using image J software. **c** Representative western blots for pEGFR/EGFR and pHER2/HER2 in therapy sensitive (SKBR3) or resistant (Gef R, LapR variants, maintained in the indicated concentrations of gefitinib or lapatinib. **d** Fold change in EGFR expression, **e** Fold change in HER2 expression, **f** Fold change in EGFR phosphorylation, **g** Fold change in HER2 phosphorylation, *N* = 3 ± SEM. **** = *p* ≤ 0.001, *** = *p* ≤ 0.005, ** = *p* ≤ 0.01, * = *p* ≤ 0.05

To determine the effectiveness of anti-EGFR therapy in the therapy sensitive and resistant variants, we evaluated the levels of EGFR and HER2 and their activation (phospho (p)-EGFR and p-HER2) in the therapy sensitive and resistant cells exposed to the same concentrations of gefitinb and lapatinib used to create the therapy resistant variants. As expected, gefitinib reduced the phosphorylation of EGFR in sensitive SKBR3 cells at 0.1 μM and 0.5 μM concentrations (Fig. 2A, B). Even though gefitinib was developed to interact only with the ATP domain of EGFR, our results show that gefitinib also significantly decreased HER2 phosphorylation by 50–70% in a concentration dependent manner. Notably, the expression of total EGFR and HER2 was significantly elevated following 24 h in 0.5 μM gefitinib and 0.1 μM lapatinib treatments even in the sensitive SKBR3 cells, suggesting a possible mechanism of compensation (Fig. 2B). The cell variants resistant to geftinib 0.1 μM and lapatinib 0.1 μM continued to respond to the drugs by decreased pEGFR and pHER2 levels demonstrating that as expected, the TKIs continued to act by inhibition of receptor phosphorylation (Fig. 2C). Of note are the SKBR3 Lap. R cells, which demonstrated increased EGFR expression compared to the sensitive cells, also suggesting a mechanism to compensate the decrease in activation (Fig. 2D). However, Gef. R cells demonstrated no changes in expression of EGFR or HER2 (Fig. 2 C-E). The cells resistant to 0.5 μM gefitinib demonstrated sustained phosphorylation of EGFR, suggesting a different mechanism of resistance than in the cells exposed to lower concentrations of gefitinib (Fig. 2F). Although gefitinib and lapatinib continued to inhibit EGFR and HER2 phosphorylation, and thus activation, these therapeutics did not affect the viability of the Gef. R and Lap. R cells, suggesting alternate mechanisms (Fig. 1).

### Effect of EGFR therapy on apoptosis in therapy resistant breast cancer cells

**Fig. 3:**
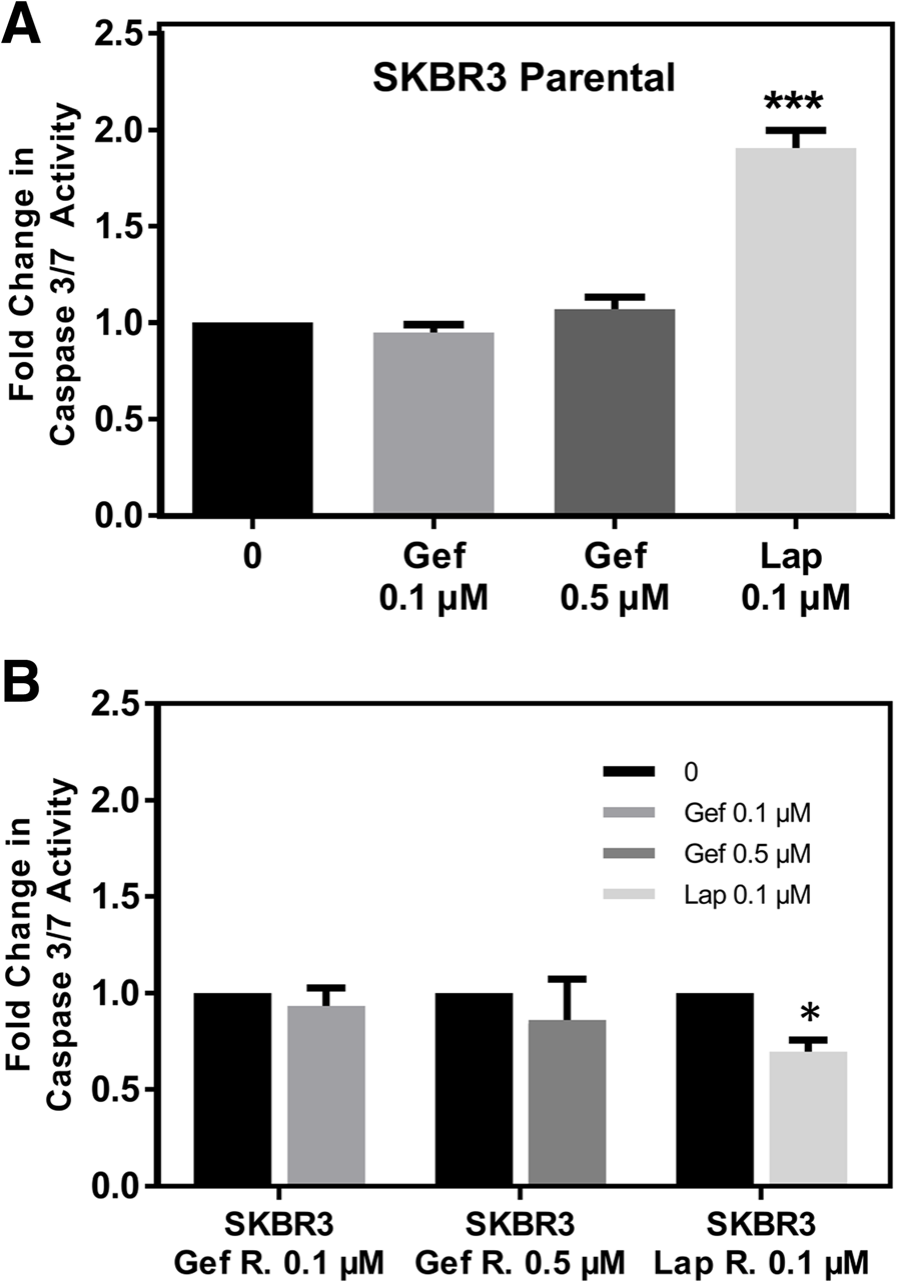
Apoptosis in therapy sensitive and resistant variants Apoptosis in therapy sensitive and resistant SKBR3 cell variants was detected by a Caspase-Glo 3/7 Assay. **a** Fold change in Caspase 3/7 activity in the therapy sensitive SKBR3 cell line following Gef or Lap treatment for 48 h compared to the vehicle controls. **b** Fold change in caspase 3/7 activity in the therapy resistant cell lines following treatment compared to non-treated cells. *N* = 3 ± SEM, * = *p* ≤ 0.05, *** = *p* ≤ 0.005

Previous studies have shown that lapatinib induces apoptosis in breast cancer cells [49]. In order to test the hypothesis that lapatinib no longer induces apoptosis in the therapy resistant cell lines, we performed a Caspase-Glo 3/7 assay. As expected, the sensitive SKBR3 cells did not respond to gefitinib by apoptosis, but exhibited a 2-fold higher statistically significant increase in caspase 3/7 activity in response to 0.1 μM lapatinib, when compared to vehicle control (Fig. 3A). However, the lapatinib resistant variant showed a significant decrease in caspase 3/7 activity in response to lapatinib (Fig. 3B), suggesting that these cells are not only resistant to the treatment, but in the presence of the treatment, resistant cells may create an optimal environment for evading apoptosis.

### Mammosphere forming efficiency of therapy resistant breast cancer cells

Since cancer stem cells (CSCs) are an integral part of tumor progression, certain therapeutics can enrich the CSC population during acquisition of therapy resistance. Moreover, researchers have found that these CSCs share properties with metastatic cancer cells essential for providing a tumor microenvironment to support the growth of metastatic cells, along with evasion of cell death and increased survival [50]. Additionally, the CSC hypothesis sustains that since normal stem cells tend to be quiescent, dormant CSCs may be resistant to therapies that target dividing cells [51].

**Fig. 4:**
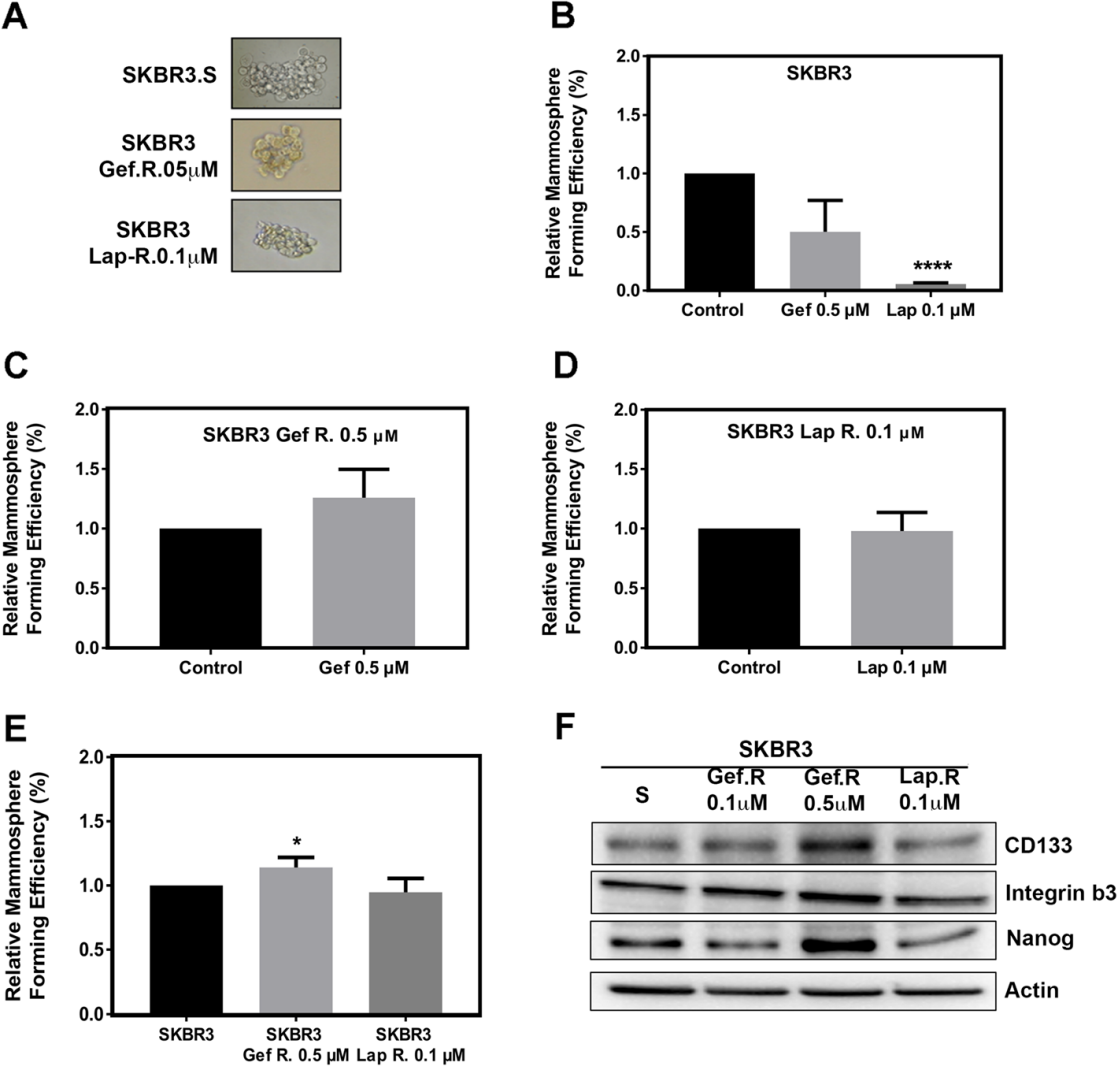
Stem cell-like characteristics in therapy resistant variants. Mammosphere formation efficiency **(**MFE) of SKBR3 therapy sensitive and resistant variants was calculated by dividing the number of mammospheres formed by the number of cells seeded per well and multiplied by 100 for percentage. **a** Representative micrographs of mammosphere forming units. Fold changes of percentage are shown in: **b** MFE in gefitinib and lapatinib treated therapy sensitive cells relative to vehicle treated cells. **c,d** MFE in therapy resistant cells treated with **(c)** gefitinib or **(d)** lapatinib, relative to vehicle controls. **e** MFE of therapy resistant variants relative to therapy sensitive cells with no treatment. **f** Representative western blots of cancer stem cell markers integrin β3, CD133, and Nanog in SKBR3 therapy sensitive and resistant variants. *N* = 3 ± SEM,* = *p* ≤ 0.05 and,**** = *p* ≤ 0.001

Therefore, to determine if the therapy resistant cells include a higher percentage of stem cell-like cells, a mammosphere assay was performed, as in [52, 53]. Therapy sensitive SKBR3 cells showed a significant reduction in mammosphere formation after treatment with 0.5 μM gefitinib or 0.1 μM lapatinib (Fig. 4B). However, treatment with gefitinib or lapatinib had no significant effect on mammosphere formation in the therapy resistant variants (Fig. 4C, D). Moreover, SKBR3 Gef. R cells resistant to 0.5 μM gefitinib showed a significant increase in mammosphere formation, and a correlative increase in the expression of stem cell markers such as integrin β3, CD133, and Nanog (Fig. 4E and F). This result suggests that higher concentrations of gefitinib may be inducing different mechanisms of resistance and may provide a better environment for the survival and promotion of a stem cell-like phenotype in therapy resistant cells.

### Molecular mechanisms of EGFR therapy resistance in breast cancer cells

**Fig. 5:**
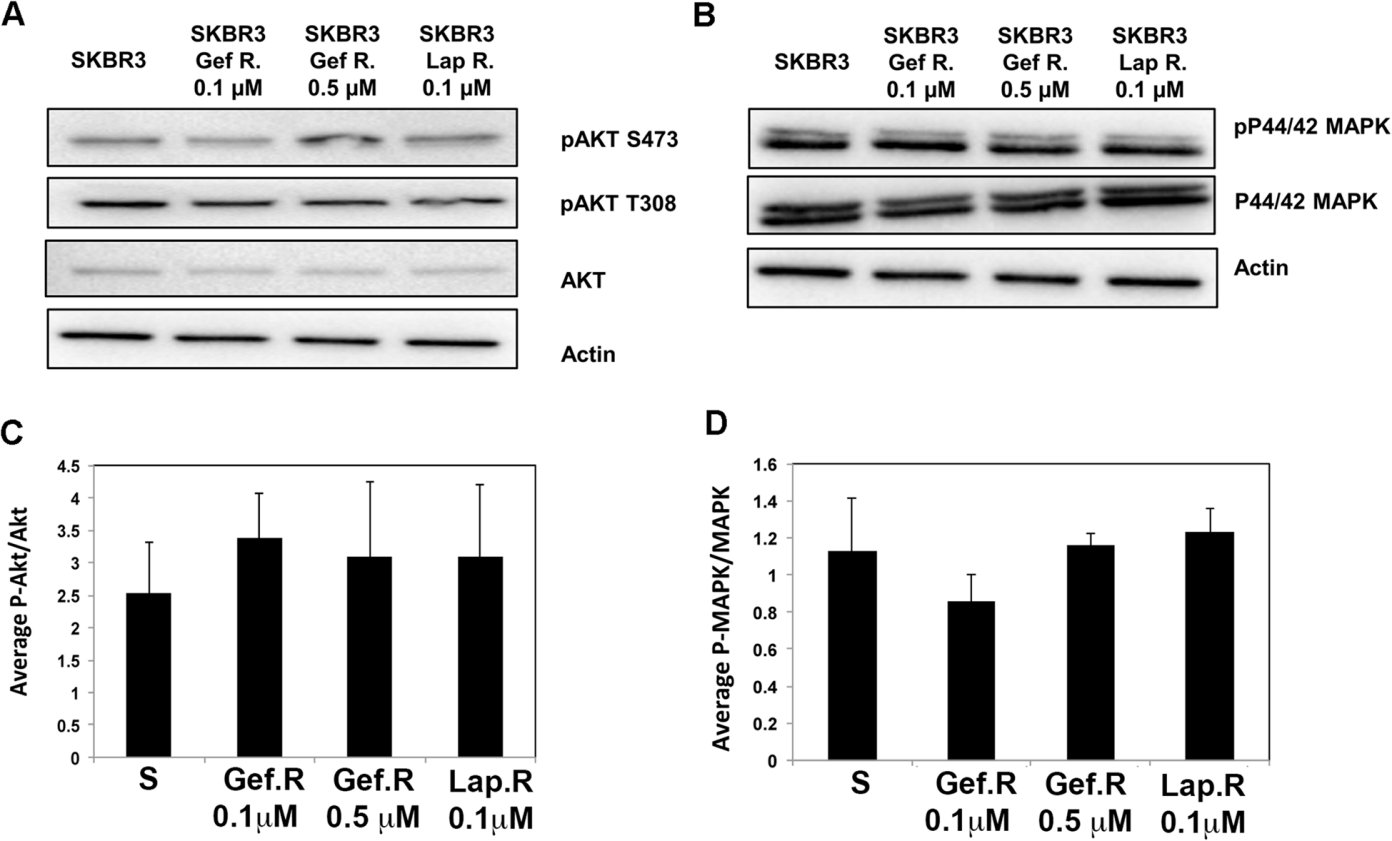
Akt and MAPK activities in therapy resistant variants. SKBR3 gefitinib and lapatinib sensitive and resistant cells were lysed and subjected to western blotting for expression and activity of a Akt/p-Akt^S473, T308^, **b** p44/42 MAPK/p-MAPK^T202, Y204^ using total or phospho-specific antibodies to the active sites. **c**, **d** Average integrated density of p-Akt/Akt (**c**) or p-P44/42 MAPK/P44/42 MAPK (**d**), as quantified from Image J analysis of positive bands from western blots. *N* = 3

EGFR/HER2 therapy resistance is often due to upregulation of downstream signaling via phosphoinositide 3-kinase (PI3-K)/Akt, Ras/mitogen activated protein kinase (MAPK) or Rac/Cdc42/p21-activated kinase (PAK) pathways [13, 15, 26, 33, 54, 55]. Therefore, we tested the levels of expression and activation of AKT and MAPK in the therapy resistant cells compared to the therapy sensitive SKBR3 cell line, using antibodies to total and phospho (active) proteins. However, no significant changes were observed in the expression or activation of Akt or p42/44 MAPK in the therapy resistant variants compared to the therapy sensitive cell line (Fig. 5).

**Fig. 6:**
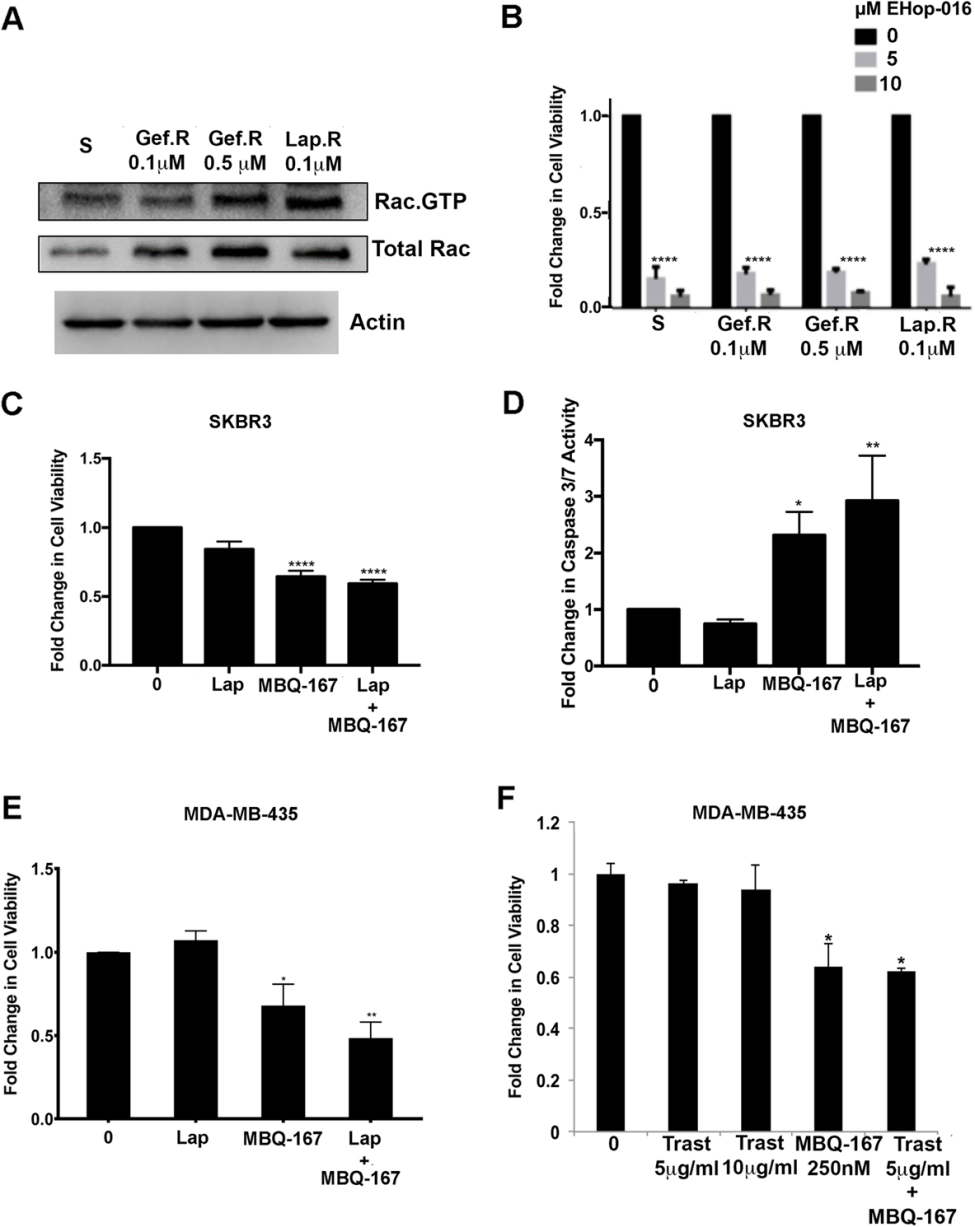
Inhibition of upregulated Rac in therapy resistant variants. **a** Rac activation was determined by a pulldown assay using the p21-binding domain of p21-activated kinase (PAK) from lysates of therapy sensitive or resistant SKBR3 cells. Representative western blots for active Rac.GTP, total Rac, and actin are shown. **b** SKBR3 gefitinb and lapatinib sensitive and resistant cells were subjected to a MTT assay for cell viability following 24 h in the Rac inhibitor EHop-016 at 0, 5, or 10 μM. **c** SKBR3 lapatinib resistant cells were subjected to a MTT assay for cell viability following 24 h in vehicle (0), 0.1 μM lapatinib, 250 nM MBQ-167, or a combination of 0.1 μM lapatinib and 250 nM MBQ-167. **d** SKBR3 lapatinib resistant cells were subjected to a caspase3/7 assay for apoptosis following 24 h in vehicle (0), 0.1 μM lapatinib, 250 nM MBQ-167, or a combination of 0.1 μM lapatinib and 250 nM MBQ-167. **e** MDA-MB-435 laptinib resistant HER2+ cells were treated with 0.1 μM lapatinib, 250 nM MBQ-167, or a combination of 0.1 μM lapatinib and 250 nM MBQ-167 for 48 h and cell viability quantified by a MTT assay; fold change in cell viability relative to vehicle is shown. **f** MDA-MB-435 trastuzumab resistant HER2+ cells were treated with 5 or 10 μg/ml trastuzumab, 250 nM MBQ-167, or a combination of 5 μg/ml trastuzumab and 250 nM MBQ-167 for 48 h and cell viability quantified by a MTT assay; fold change in cell viability relative to vehicle is shown. *N* = 3 ± SEM * = *p* ≤ 0.05, ** = *p* ≤ 0.01**** = *p* ≤ 0.001

Since the Rho GTPase Rac signaling downstream of EGFR and HER2 have been shown to contribute to EGFR/HER2 therapy resistance [38, 42, 43, 56–58], we performed expression and activation assays to determine the role of Rho GTPases in the therapy resistant variants. Notably, compared to the therapy sensitive SKBR3 cell line, the therapy resistant cells demonstrated increased Rac expression, and thus, enhanced Rac activity (Fig. 6A). Moreover, no significant changes in expression were observed for the related Rho GTPases Rho and Cdc42 (Data not shown). To determine whether the increased Rac activation contributed to therapy resistance, we tested the effect of the Rac inhibitor EHop-016 [46] in therapy sensitive and resistant SKBR3 cells. Results show a statistically significant decrease in cell viability at 5 and 10 μM EHop-016 for both sensitive and resistant cell variants (Fig. 6B).

We next tested an additional Rac inhibitor MBQ-167 that we recently developed and characterized as a more potent Rac and Cdc42 inhibitor compared to EHop-016 [25, 46] in lapatinib resistant SKBR3 cells. Results show that while lapatinib did not affect the viability of the resistant variant, 0.5 μM MBQ-167 alone or in combination with 0.5 μM lapatinib significantly decreased cell viability by ~ 40% (Fig. 6C). This reduction in cell viability resulted in apoptosis as seen by > 2-fold increase in caspase 3/7 activity following MBQ-167 (0.25 μM) and an even higher significant increase in caspase activity when MBQ-167 (0.25 μM) was administered in combination with lapatinib (0.5 μM) (Fig. 6D). The gefitinib resistant SKBR3 variants also responded to the Rac inhibitor MBQ-167 by a similar phenotype of cell rounding, detachment from the substrate, and subsequent death, as we have reported in [46] (Supplemental Fig. 3).

To determine if this is a universal mechanism of resistance, we determined the effect of Rac inhibition in a highly metastatic and therapy resistant variant of the MDA-MB-435 cell line, which we have previously shown to demonstrate upregulated Rac compared to its less metastatic variants [47]. As shown in Fig. 6 E,F, the metastatic MDA-MB-435 variant is insensitive to lapatinib and trastuzumab, a monoclonal antibody to the HER2 receptor, which is overexpressed in this cell line. However, the Rac/Cdc42 inhibitor MBQ-167 decreased the viability of this cell line by ~ 40%. Combined lapatinib and MBQ-167 decreased cell viability further by ~ 50%. MBQ-167 also inhibits MDA-MB-435 cell viability in the presence of trastuzumab, thus demonstrating its potential to inhibit therapy resistant cell viability. Thus, this data implicates Rac activation in EGFR/HER2 therapy resistance, and the potential of direct Rac inhibition by small molecule inhibitors to overcome TKI therapy resistance.

## Discussion

The EGFR (ErbB) family members are central transducers of a myriad of cellular signaling cascades that drive cancer progression [55]. Specifically, the EGFR type II (HER2) may heterodimerize with the other three members of the family (EGFR1, EGFR3 and EGFR4) coordinating a series of pathways that lead to cell survival, proliferation, and invasion/migration [59]. The overexpression of EGFR family members has been observed in more than 20% of invasive breast carcinomas and this amplification is associated with increased metastatic potential. Therefore, anti-EGFR therapy is considered a viable targeted strategy for cancers that overexpress these receptors. The use of lapatinib, a dual EGFR/HER2 therapeutic, has improved breast cancer patient survival when used in combination with HER2-targeted therapeutics such as trastuzumab [60]. However, the failure in the approval of gefitinib, and the resistance by many patients to trastuzumab and lapatinib, remains a challenge in using these therapeutics [61–64] . Therefore, the identification of resistance pathways and the development of new approaches to enhance patient response to TKIs is a critical objective, where combination therapy targeting the downstream signaling pathways is a viable strategy [65].

For this study, clinically relevant therapy resistant syngenic variants were successfully created from the SKBR3 therapy sensitive breast cancer cell line, and used as a model to investigate the mechanisms of resistance to both gefitinib and lapatinib. As observed, anti-EGFR therapy continues to inhibit EGFR and HER2 phosphorylation in the therapy resistant cells similar to the therapy sensitive cells. Interestingly, resistant cells that were exposed to the higher concentration (0.5 μM) of gefitinib did not respond via direct inhibition of EGFR or HER2 phosphorylation. This may be due to the acquisition of a resistant mutation, such as the EGFR T790M secondary mutation, which results in insensitivity to EGFR targeted therapy [66]. In addition, the expression levels of EGFR and HER2 were higher in the therapy sensitive cells following TKI treatments, as well as in the lapatinib resistant cells (for EGFR), indicating that these cells may be synthesizing more receptors to compensate for the inactivation of this pathway. Also, even though it has been shown that gefitinib is a specific inhibitor of the tyrosine kinase domain of EGFR, our data shows that gefitinib also decreases the phosphorylation of HER2. These effects on HER2 activity may be related to the heterodimerization complexes that occur between receptors (e.g. EGFR1 and HER2), which can lead to a decrease in protein phosphorylation of both subunits in response to gefitinib.

Lapatinib treatment has been shown to induce apoptosis in trastuzumab-resistant breast cancer cells [67]. Therefore, as expected, lapatinib induced apoptosis in SKBR3 therapy sensitive cell lines; however, the therapy resistant cells evade apoptosis in the presence of the treatment suggesting that not only are these cells resistant to the treatments, but prolonged therapy provides an environment optimal for avoiding apoptosis. Even though gefitinib has been shown to induce apoptosis in other cancer cell types, including breast cancer, the SKBR3 cells did not respond to gefitinib treatment via apoptosis. This has also been confirmed by other studies where the apoptotic response to gefitinb was cell type-dependent [68, 69]. This lack of response maybe because autophagy and not apoptosis has been shown to be an early response to gefitinib treatment in SKBR3 cells [70].

In addition to evasion of apoptosis, cancer cells undergo EMT during metastatic progression, which may produce subpopulations of cells with stem cell-like characteristics that contribute to therapy resistance [71]. As expected, the SKBR3 therapy sensitive cells respond to gefitinib or lapatinib treatment with lower MFE used as a measure of stem cell-like activities, whereas TKI treatment had no effect in the therapy resistant cells. Moreover, we observed an increase in MFE and established breast cancer stem cell markers in cells resistant to the higher concentration of gefitinib, suggesting that the therapy resistant breast cancer cells may have more cancer stem cell activity that can contribute to therapy resistance.

Similar to trastuzumab, lapatinib resistance results in circumvention of the kinase inhibitory function by acquiring point mutations in HER2 and EGFR, as well as via elevated downstream signaling [72–75]. Therefore, activation of compensatory pathways downstream of EGFR and HER2 is a common mechanism of resistance to lapatinib and gefitinib therapy. Central to these pathways are the activation of Akt via PI-3 K and the Ras/MAPK pathway [15, 76]. However, when investigating potential mechanisms of therapy resistance and the possible activation of compensatory pathways in our study, we found that Akt and MAPK activities (Phosphorylation) were unchanged in the therapy resistant SKBR3 cells.

Of note is the finding that expression and activity of the Rho GTPase Rac, but not related family members RhoA and Cdc42, are elevated in the therapy resistant variants. The Rho GTPase family is known to regulate therapy resistance and CSC maintenance [77, 78]. Of the Rho GTPases, Rac has been implicated with cancer therapy resistance, specifically via the oncogenic guanine nucleotide exchange factors that are coupled to EGFR and HER2 signaling. Numerous studies have implicated Rac/PAK activities with the maintenance of mesenchymal stem cell-like populations in epithelial cancers; and thus, therapy resistance, especially in HER2-type breast cancer [33, 36, 38–43, 79–86]. Accordingly, our results with the Rac inhibitors EHop-016 and MBQ-167 show that both these inhibitors significantly reduce the MFE of HER2+ and EGFR+ breast cancer cells [44, 46]. Moreover, The Cancer Genome Atlas (TCGA) data show that Rac1 or PAK1 overexpression is associated with malignant breast cancer and significantly diminishes HER2 type patient survival within 10 years following diagnosis [87]. Similar to our finding that Rac1 is overexpressed in therapy resistant variants of breast cancer cells, Rac1 has also been shown to be overexpressed in naturally occurring lapatinib-resistant HER2 type breast cancer cell lines [88]. Therefore, we posit that Rac1 inhibition is a rational strategy for sensitization of lapatinib and gefitinib resistant tumors.

Accordingly, in the therapy resistant variants created in this study, the Rac inhibitor EHop-016, which was designed and developed by us to inhibit Rac activation by the oncogene Vav, which is activated by EGFR/HER2 [44], or the dual Rac1/Cdc42 inhibitor MBQ-167 [46], reduced viability and induced apoptosis in single or combined treatments with lapatinib or trastuzumab. Even though there was a trend in further reduction of cell viability when the Rac inhibitor was combined with gefitinib, lapatinib, or trastuzumab in the therapy resistant variants, this effect was not additive or synergistic. However, our data clearly show the utility of using Rac inhibitors as a valid strategy to reduce viability of highly aggressive breast cancer cells. Accordingly, we have shown that in a mouse model of metastasis, the highly metastatic and therapy resistant MDA-MB-435 variant used in this study, reduced mammary fat pad tumor growth by ~ 85% and metastasis by 100% [57].

In support of a role for Rac inhibition in chemosensitization, Rac1 knockdown has been shown to sensitize lapatinib resistance [88], and a small molecule inhibitor of Rac1, NSC23766, was shown to increase sensitivity to the anti-HER2 therapeutic trastuzumab [33], overcome gefitinib resistance in non-small cell lung carcinoma [89], and be effective in combination therapy with eroltinib, another tyrosine kinase inhibitor [90]. Additionally, EHop-016 sensitizes HER2 overexpressing trastuzumab sensitive and resistant breast cancer cells to trastuzumab [44, 91–93], and was recently shown to overcome therapy resistance by combined cancer therapy with Akt/mTOR inhibitors [94]. Therefore, targeting Rac is considered a viable strategy to overcome anti-EGFR/HER2 therapy resistance in cancer [24, 25, 33, 84, 88, 89, 95].

The salient observation that the therapy resistant variants overexpress and activate Rac1, an established driver of metastasis, is highly relevant towards novel therapeutic strategies to overcome therapy resistance. Most studies illustrating the utility of Rac inhibitors have used the Tiam1/Rac inhibitor NSC23766, which is active at 50–100 μM concentrations that are too high to be pharmacologically useful [89]. In this study, we tested our patented drugs that act through disparate mechanisms: the Vav/Rac inhibitor EHop-016 and the nucleotide association inhibitor MBQ-167, at 100X lower effective concentrations than NSC23766. We attempted to establish mouse mammary tumors with the SKBR3 sensitive and resistant cell lines in immunocompromised mice but did not get tumor take, since the SKBR3 cell line does not form tumors readily in mouse models. Even though this study was conducted in vitro, we have tested EHop-016 and MBQ-167 in mouse models of HER2+ breast cancer, and have demonstrated their utility as metastasis inhibitors [44, 46, 93]. Therefore, these results signify the importance of Rac and its close homology Cdc42 as viable targets to treat therapy resistant cancer.

## Conclusions

In conclusion, malignant cancer cells hijack alternate pathways to survive anti-EGFR/HER2 therapy and grow and migrate or stay dormant. The data presented here suggests that Rac plays an integral role in the activation of EGFR/HER2 signaling during therapy resistance and that this increase in active Rac levels may promote cancer stem cell maintenance, as well as cell growth and survival. Therefore, novel therapies targeting Rac, such as EHop-016 and MBQ-167, may be potential therapeutics to use individually or in combination in therapy resistant breast cancer.

## Supplementary Information

**Additional file 1: Figure S2.** EGFR and HER2 expression and phosphorylation in therapy sensitive and resistant variants. **Figure S3.** Effect of MBQ-167 on viability of gefitinib resistant cells. **Figure S4A.** Representative western blots of cancer stem cell markers integrin β3, CD133, and Nanog in parental SKBR3 cells and the therapy resistant variants. **Figure S4F.** Representative western blots of cancer stem cell markers integrin β3, CD133, and Nanog in parental SKBR3 cells and the therapy resistant variants. **Figures S5.** a,b Akt and MAPK activities in therapy resistant variants. **Figure S6.** Rac upregulation in therapy resistant variants.

## Data Availability

The datasets used and/or analyzed during the current study are available from the corresponding author on reasonable request.

## Abbreviations

CSC: Cancer stem cell
EMT: Epithelial to mesenchymal transition
EGFR: Epidermal growth receptor
Gef.R: Gefitinib resistant
Lap.R: Lapatinib resistant
HER2: Human epidermal growth factor receptor
IC_50_: Half maximal inhibitory concentration
MAPK: Mitogen activated protein kinase
MFE: Mammosphere forming efficiency
mTOR: Mammalian target of rapamycin
MTT: 3-(4,5-dymethyl thiazol-2-yl)-2,5-diphenyl tetrazolium bromide
p: Phospho
PAK: p21-activated kinase
PI3K: Phosphoinositide 3-kinase
TCGA: The Cancer Genome Atlas
TKI: Tyrosine kinase inhibitor
