# Supplementary material for: Rac inhibition as a novel therapeutic strategy for EGFR/HER2 targeted therapy resistant breast cancer
Source: BMC Cancer. 2021 Jun 1;21:652. doi: 10.1186/s12885-021-08366-7 (PMC8170972; doi:10.1186/s12885-021-08366-7)
Supplement: Supplementary file 1 — Additional file 1: Figure S2. EGFR and HER2 expression and phosphorylation in therapy sensitive and resistant variants. Figure S3. Effect of MBQ-167 on viability of gefitinib resistant cells. Figure S4A. Representative western blots of cancer stem cell markers integrin β3, CD133, and Nanog in parental SKBR3 cells and the therapy resistant variants. Figure S4F. Representative western blots of cancer stem cell markers integrin β3, CD133, and Nanog in parental SKBR3 cells and the therapy resistant variants. Figures S5. a,b Akt and MAPK activities in therapy resistant variants. Figure S6. Rac upregulation in therapy resistant variants. [file 12885_2021_8366_MOESM1_ESM.pdf]

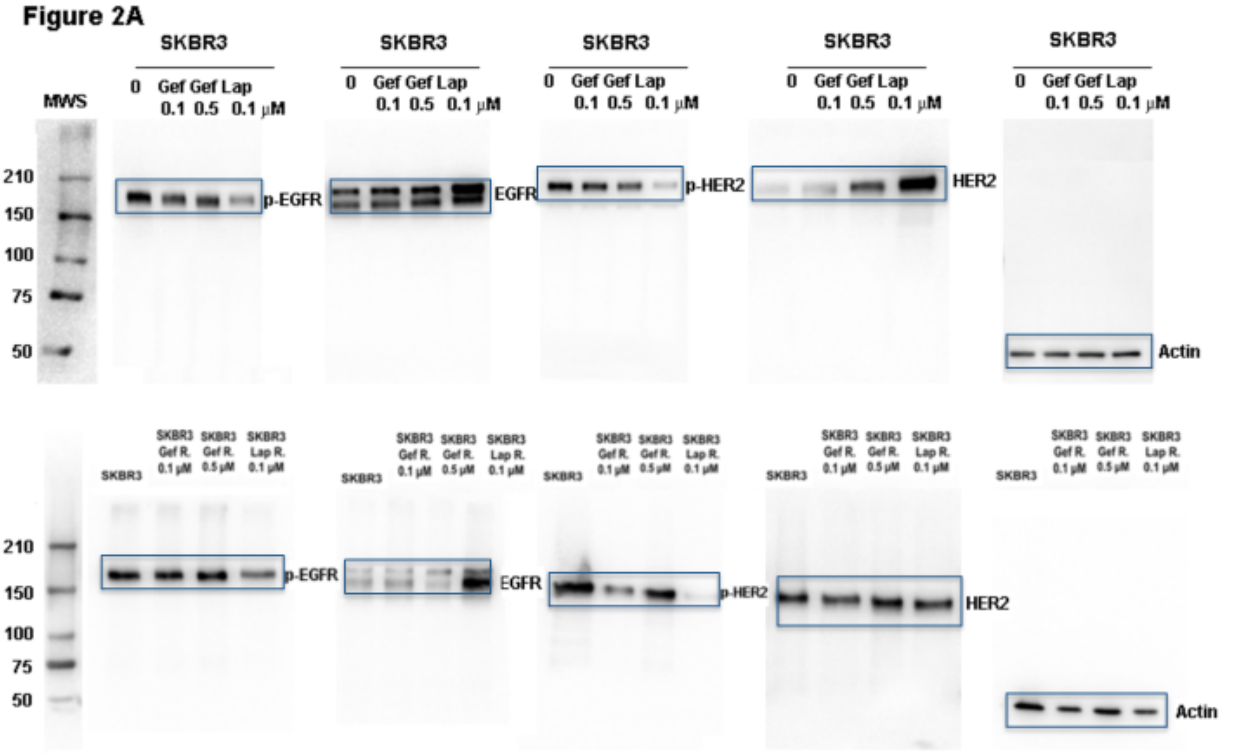

Supplemental Figure to Figure 2. EGFR and HER2 expression and phosphorylation in therapy sensitive and resistant variants. SKBR3 therapy sensitive or resistant (Gef.R 0.1 μM, Gef.R 0.5 μM and Lap.R 0.1 μM) cells treated with gefitinib or lapatinib for 24h were lysed and western blotted for total and active (phospho) EGFR and HER2. **a** Representative western blots for pEGFR/EGFR (left) and pHER2/HER2 (right), with actin as a loading control, for cells treated with gefitinib or lapatinib for 24 h.

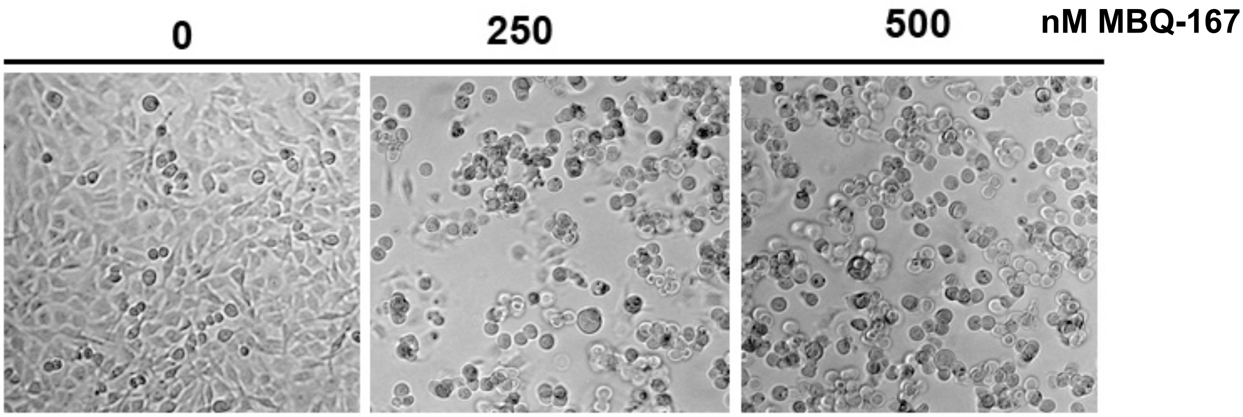

Supplemental Figure to Figure 3. Effect of MBQ-167 on viability of gefitinib resistant cells. Confluent SKBR3.Gef 0.1 μM resistant cells were treated with 0, 250, or 500 nM MBQ-167 for 24h and imaged for cell response. Note, cell rounding and anoikis in response to MBQ-167. 100X magnification.

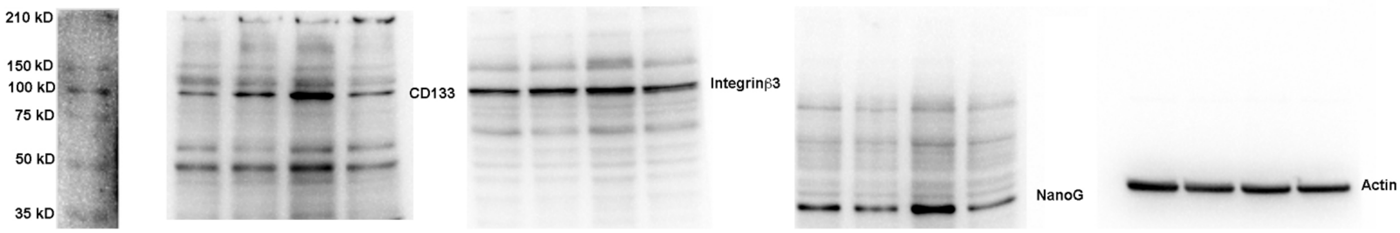

Supplemental Figure to Figure 4A. Representative western blots of cancer stem cell markers integrin β3, CD133, and Nanog in parental SKBR3 cells and the therapy resistant variants.

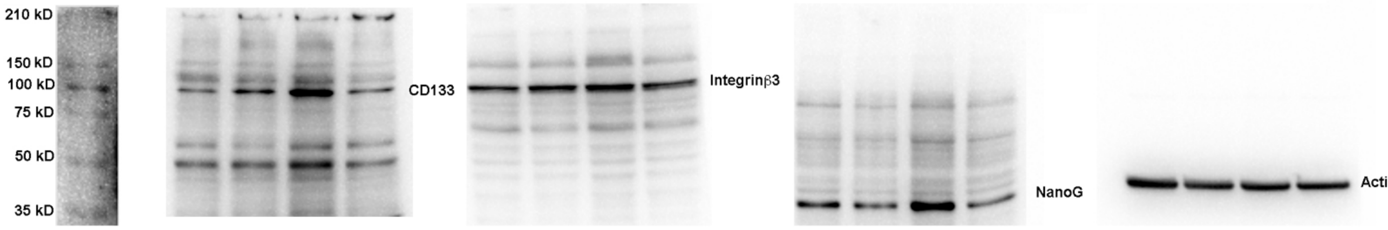

Supplemental Figure to Figure 4F. Representative western blots of cancer stem cell markers integrin β3, CD133, and Nanog in parental SKBR3 cells and the therapy resistant variants.

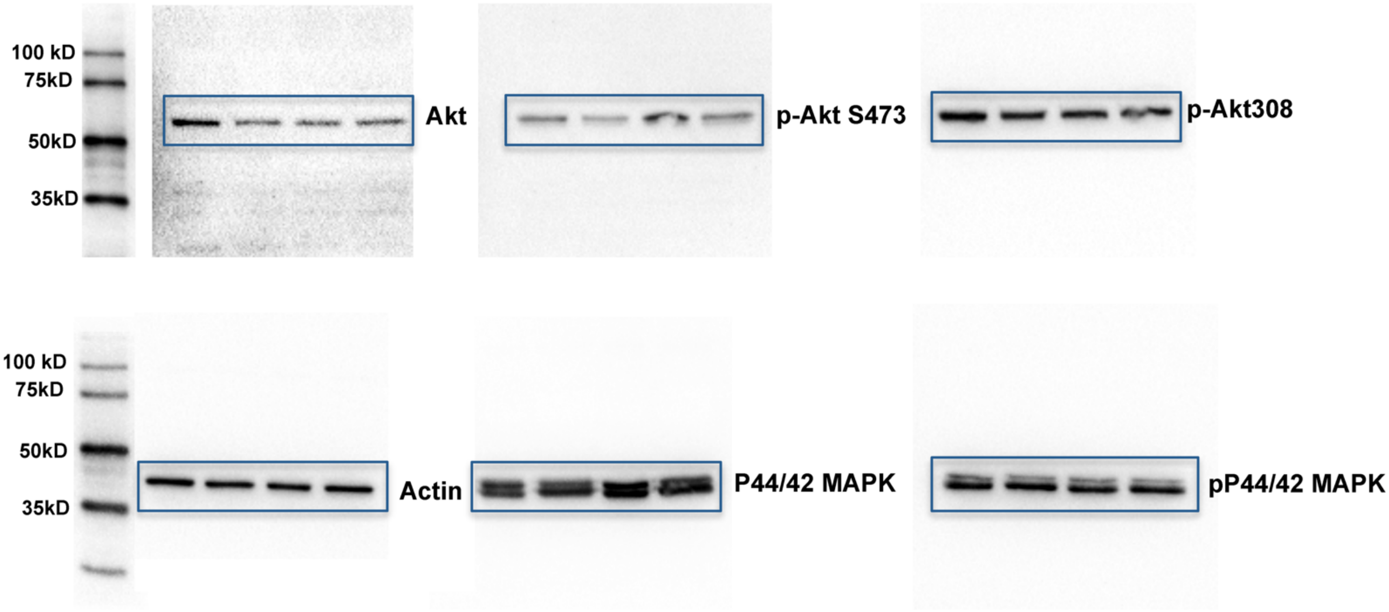

Supplemental Figure to Figures 5 a,b Akt and MAPK activities in therapy resistant variants. SKBR3 parental and the gefitinib and lapatinib resistant cells were lysed and subjected to western blotting for expression and activity of **a** Akt/p-Akt$^{S473, T308}$, **b** p44/42 MAPK/p-MAPK$^{T202, Y204}$ using total or phospho-specific antibodies to the active sites.

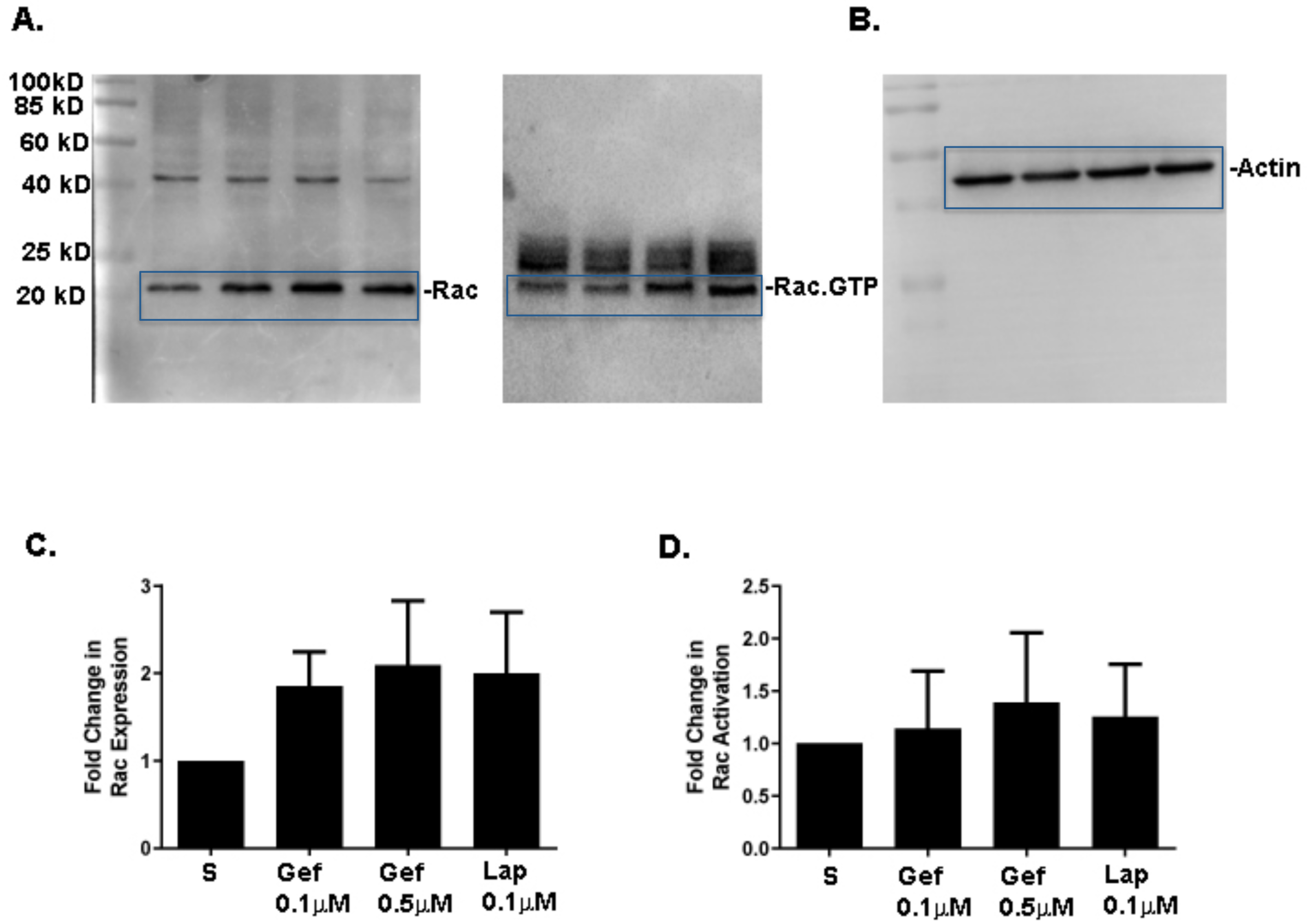

Supplemental Figure to Figure 6. Rac upregulation in therapy resistant variants. Rac activation was determined by a pulldown assay using the p21-binding domain of p21-activated kinase (PAK) from lysates of parental or therapy resistant SKBR3 cells. (A) Representative western blots for active Rac.GTP and total Rac and (B) for actin are shown. Fold change in Rac expression (C) and Rac activation (D) in the therapy resistant variants compared to the therapy sensitive parental cell line.
